# Spread of Infectious Disease.—I

**Published:** 1904-05-28

**Authors:** J. E. R. Stephens

**Affiliations:** Author of "Digest of Public Health, Case-Law," etc.


### HOSPITAL ADMINISTRATION.

### CONSTRUCTION AND ECONOMICS.

### SPREAD OF INFECTIOUS DISEASE.—I.

By J. E. R. STEPHENS, Barrister-at-Law, Author of "Digest of Public Health, Case-Law," etc.

It is interesting to observe, in spite of the common belief that all sanitary precautions in the case of infected persons are of modern growth, that the history of the law upon the subject of safeguarding the public against the spread of infectious disorders reaches far back into the past. Sir Anthony Fitzherbert, who was a judge of the Court of Common Bench in the reign of King Henry VIII., describes in his treatise, "De Natura Brevium," 234, a writ called *de leproso amovendo*, which lies, he says, where a man is a lazar or leper, and is dwelling in any town, and he will come into the church or amongst his neighbours where they are assembled, to talk with them, to their annoyance and disturbance. That writ directed the sheriff to have the man examined, and if found to be a leper to cause him to be carried away to a solitary place, to dwell there as the custom is, lest by such his common conversation, damage or peril should in anywise happen to his neighbours. "But it seemeth," adds the learned judge, "that if a man be a leper or a lazar, and will not converse with his neighbours, then he shall not be moved out of his house." The scourge of leprosy was succeeded in after years by the scourge of the plague, and by a statute of James I. (1 Jac. I. c. 31, sect. 7), any person infected with the plague might be commanded by the corporate officers of a town or by a justice or constable in a county to keep his house, for avoiding of further infection, and might be forcibly kept in; and if he, nevertheless, got abroad, and conversed in company, having any infectious sore upon him uncured, he might be punished as a felon, but if no such sore was found upon him, he might be punished as a vagabond. This statute was a temporary one, and Sir Mathew Hale, writing in the reign of King Charles II., says that "it is now discontinued; but what if such person goes abroad to the intent to infect another, and another is thereby infected and dies? Whether this be not murder by the common law might be a question, but if no such intention evidently appear, though *de facto* by his conversation another be infected, it is no felony by the common law, though it be a great misdemeanour." Notwithstanding what is said by Sir Mathew Hale, Mr. Serjeant Hawkins, in his "Pleas of the Crown," cites the statute of James as if it were in full force, in treating of offences against public health, which he classifies as of two kinds, viz.: (1) spreading the plague, and (2) neglecting quarantine, as prescribed by 26 Geo. II., c. 6. However, the plague, like leprosy, gradually seems to have died out, and was succeeded in turn by a new public terror, the small-pox.

#### SMALL-POX INFECTION.

In 1752 a motion was made before Lord Chancellor Hardwicke for an injunction to stay the building of a house to inoculate for small-pox in Cold Bath Fields. Lord Hardwicke refused to grant an injunction upon the ground that such a house was not a public nuisance, and if it had been the proper remedy was by information. The question came up for discussion in the year 1815, in *Rex* v. *Vantandillo* (4 M. & S. 73). There the defendant was indicted for carrying her child, while infected with small-pox, along a public highway, and having suffered judgment to go by default, appeared to receive sentence, but moved in arrest of judgment that the indictment disclosed no offence known to the law. The Court thought otherwise, and Mr. Justice Le Blanc in passing sentence observed that, although the Court had not found upon its records any prosecution for this specific offence, yet there could be no doubt in point of law that if a person unlawfully, injuriously, and with full knowledge of the fact, exposes in a public highway a person infected with a contagious disorder, it is a common nuisance to all the subjects, and indictable as such. The Court did not pronounce that every person who inoculated for this disease was guilty of an offence, provided it was done in a proper manner, and the patient was kept from the society of others, so as not to endanger a communication of the disease. In such a case the law did not pronounce it to be an offence. But no person having a disorder of this description upon him ought to be publicly exposed to endangering the health and lives of the rest of the subjects. In the same year another case occurred, that of *Rex* v. *Burnett* (4 M. & S. 272). Here an apothecary was convicted on indictment for having, after inoculating some children with the small-pox, incautiously exposed them, while infected with the disease, in the public street, to the danger of the public health.

The present statute law on the subject is contained in Sect. 126 of the Public Health Act, 1875, and Sects. 68 and 70 of the Public Health (London) Act, 1891; as to Scotland in Sects. 48 and 49 of the Public Health (Scotland) Act 1867; and as to Ireland in Sect. 142 of the Public Health (Ireland) Act 1871.

The leading case in England is that of *Tunbridge Wells Local Board* v. *Bisshopp* (2 C. P. D. 187). In this case a medical man in practice in Tunbridge Wells sent a patient who was suffering from scarlet fever to the fever hospital there with a certificate, directing him to walk in the middle of the road, and not to talk to anyone; but, in consequence of an alleged informality in the certificate, the patient was refused admission, whereupon the medical man walked with him through the streets of the town to the residence of the chairman of the local board, from whom after some delay he obtained an order for the man's admission to the hospital. He then returned with the patient to the police station to procure the ambulance to convey him thither. Upon an information against the medical man for an alleged infringement of the statute, the justices were of opinion that it was not proved before them that the medical man "had charge of" the patient; that he had not wilfully exposed the patient in any street or public place "without proper precaution"; and that he had made the best use of the means at his disposal to prevent the spread of the fever. and they refused to convict him. It was held that their decision was right.

In 1894 there was a case tried in Scotland—namely, that of *Malloch* v. *Hunter* (31 Sc. L. R. 332). In that case a medical practitioner was charged with a contravention of Sect. 49 of the Public Health (Scotland) Act, 1867, in that "he being the person in charge of A. B.," a person suffering from enteric fever, being an infectious disorder, "did wilfully expose him while he was suffering as aforesaid from an infectious disorder, by placing him in a cab or hackney carriage, being a public conveyance," and conveying him from his lodgings to a hospital "without taking proper precautions against spreading the said disorder." It was proved that the accused had taken A. B., while suffering from enteric fever, to a hospital in a cab, being a public conveyance, furnished with leather fittings, but there was no evidence as to what other precautions against spreading the disorder were necessary. The magistrate convicted the accused. But it was held on appeal (1) that there was no offence committed under Sect. 49 unless the patient was exposed without proper precautions being taken against endangering the public safety by spreading infection; (2) that the onus lay on the prosecution to prove what precautions were necessary, and that they had not been used; and (3) that as the prosecution had failed to discharge this onus, the conviction must be quashed.

### Erection of Hospitals.

Under Sect. 131 of the Public Health Act, 1875, local authorities are empowered, but not required, to provide for the use of their district, hospitals or temporary places for the reception of the sick. In the leading case of *Metropolitan Asylums District* v. *Hill* (6 A. C. 193), decided on somewhat analogous provisions in the Metropolitan Poor Act, 1867, there Lord Blackburn thus lays down the common law, independent of statute: "Those who have the charge of a sick person, if he is helpless (whether the disease be infectious or not), are at common law under a legal obligation to do, to the best of their ability, what is necessary for the preservation of the sick person. And the sick person, if not helpless, is bound to do so for his own sake. When the disease is infectious there is a legal obligation to the sick person, and those who have the custody of him, not to do anything that can be avoided which shall tend to spread the infection, and if either do so, as by bringing the infected person into a public thoroughfare, it is an indictable offence; though it will be a defence to an indictment if it can be shown that there was a sufficient cause to excuse what is *prima facie* wrong. . . . For though I think it an incident to the use of a habitation in a town that the occupier must bear the necessary risks of the inmates of a neighbouring house falling ill of a contagious disease, I do not think it an incident that he is to submit to his neighbours wilfully, though for very laudable motives, and not maliciously, bringing in contagion where it did not previously exist, if the effect is not merely to alarm him, but injure him. By necessary inference, it follows that to gather together in one spot patients suffering from infectious disease is lawful, but it must be under such guards as not to endanger the public health by communicating this infectious disease, and, as it seems to me, so as not to produce an injury to the rights of the owners of adjoining property by producing a nuisance to it."

There is nothing in the Public Health Acts to cast upon sanitary authorities the custody of sick persons, whether paupers or not. If paupers, whether by reason of their sickness only or not, the guardians of the poor are responsible for relieving them according to their necessity. If not paupers they are bound to relieve themselves, and if they are helpless the next person in authority on the premises is responsible for not exposing them. But if the local sanitary authorities assume the custody of the patient, they at once incur grave responsibilities at common law. They are under no legal duty to interfere at all. Consequently if a local authority establish a hospital and it is from its situation or management a nuisance, however praiseworthy the motives of the authority, however personally careful their members, the authority is liable to be mulcted in damages.

### Notification of Phthisis.

It is said that in England and Wales no fewer than 60,000 persons die every year from phthisis. It is also said that the infection from this disease can be prevented, and experts in sanitary science are devising various schemes for the extermination of this disease, as they believe that it can be extinguished as thoroughly as small-pox. During the last 60 years there has been a very steady decline of deaths from phthisis. Improved sanitary conditions have therefore done much. Medical officers of health have proposed that phthisis should be added to the list of diseases which require to be notified under the Infectious Diseases (Notification) Act 1889. In some districts the local authorities have proposed to do so, but a local authority cannot include a new disease within the definition of the Act without the consent of the Local Government Board, and this consent has been absolutely refused. The reason for this refusal seems to be owing to the lengthened period over which the disease lasts, which would cause too much interference with the subject and also interfere with the patient following his occupation in life. Moreover, if the patient was isolated, employment for some years would have to be found him, and many new hospitals and sanatoria would have to be built costing enormous sums of money and involving heavy additional burdens on the ratepayers.

